# A New Medicine

**Published:** 1872-05

**Authors:** 


### A NEW MEDICINE.

THE "London Lancet" has an article on the value of flowers as a medicine, and the odor of them as a preventive against diseases which prevail in marshy districts. During the war we recommended that well dressed, handsome women, young ladies, and little girls, should pass through the hospitals where the soldiers were confined, with baskets of flowers for distribution, once or twice a week, or oftener. It had been observed that when soldiers expected visits from the ladies belonging to the Christian Commission, they were more attentive to personal appearance, tidiness, and cleanliness, in all their surroundings. The sight of a fresh flower, of the sunny face of childhood, of womanly beauty, physical or moral, will do any man good, will quicken the fires of life, and wake up expiring energies; and then how they carry the mind back to home, and sister, and mother, and wife. There is a moral medicine more potent than the drug of the apothecary, — the power over the sick of gentleness, kindness, and sympathy, on the part of youth, and beauty, and womanliness. And there is a moral poison, more deadly than arsenic, or prussic acid, or nicotine. Many a pining invalid has been hurried into the grave from the want of sympathy on the part of those who should have watched, and loved, and cherished with warmest affection, hearts that were yearning, even dying for it, for some little exhibition now and then of loving tenderness.

"Don't leave me, my son," were among the very last words of Henry Clay, as the death-drops thickly studded his noble brow; it was the instinctive want of a helping hand in the terrible hour. And so do we all in sickness pine for the presence of those nearest and dearest to us, when even holding the thin bony fingers sends a sympathetic thrill to the invalid's bosom. "Crown me with flowers," was the last aspiration of the dying Mirabeau.

The atrocious Danton, the Mirabeau of the rabble, shortly before his execution, spoke incessantly of flowers, as if the very memory of them was beautiful and happifying.

---

Cool Heads for the summer promote the circulation, and tend to prevent colds, apoplexies, and sun-stroke.

